# Natural radioactivity levels in soils, rocks and water at a mining concession of Perseus gold mine and surrounding towns in Central Region of Ghana

**DOI:** 10.1186/s40064-016-1716-5

**Published:** 2016-02-01

**Authors:** A. Faanu, O. K. Adukpo, L. Tettey-Larbi, H. Lawluvi, D. O. Kpeglo, E. O. Darko, G. Emi-Reynolds, R. A. Awudu, C. Kansaana, P. A. Amoah, A. O. Efa, A. D. Ibrahim, B. Agyeman, R. Kpodzro, L. Agyeman

**Affiliations:** Environmental Protection and Waste Management Centre, Radiation Protection Institute, Ghana Atomic Energy Commission, Legon, P. O. Box LG 80, Accra, Ghana

**Keywords:** Natural radioactivity, Gold mine, Gamma spectrometry, Gross alpha, Gross beta, Annual effective

## Abstract

Levels of naturally occurring radioactive materials prior to processing of gold ore within and around the new eastern concession area of Perseus Mining (Ghana) Limited were carried out to ascertain the baseline radioactivity levels. The study was based on situ measurements of external gamma dose rate at 1 m above ground level as well as laboratory analysis by direct gamma spectrometry to quantify the radionuclides of interest namely; ^238^U, ^232^Th and ^40^K in soil, rock, ore samples and gross alpha/beta analysis in water samples. The average absorbed dose rate in air at 1 m above sampling point using a radiation survey metre was determined to be 0.08 ± 0.02 μGyh^−1^ with a corresponding average annual effective dose calculated to be 0.093 ± 0.028 mSv. The average activity concentrations of ^238^U, ^232^Th, and ^40^K in the soil, rock, and ore samples were 65.1 ± 2.2, 71.8 ± 2.2 and 1168.3 Bqkg^−1^ respectively resulting in an average annual effective dose of 0.91 ± 0.32 mSv. The average Radium equivalent activity value was 257.8 ± 62.4 Bqkg^−1^ in the range of 136.6–340.2 Bqkg^−1^. The average values of external and internal indices were 0.7 ± 0.2 and 0.9 ± 0.2 respectively. The average gross alpha and gross beta activity concentrations in the water samples were determined to be 0.0032 ± 0.0024 and 0.0338 ± 0.0083 Bql^−1^ respectively. The total annual effective dose from the pathways considered for this study (gamma ray from the soil, rock and ore samples as well as doses determined from the gross alpha/beta activity concentration in water samples) was calculated to be 0.918 mSv. The results obtained in this study shows that the radiation levels are within the natural background radiation levels found in literature and compare well with similar studies for other countries and the total annual effective dose is below the ICRP recommended level of 1 mSv for public exposure control.

## Background

Artificial and natural radioactivities are the two main sources of radiation exposure. Human activities such as mining and mining processes, oil and gas extraction may result in situations where the radioactivity levels of materials that contain natural radionuclides are significant enough to warrant regulatory control (UNSCEAR 2000; IAEA 2005). The two main sources of natural radiation exposure are from terrestrial radionuclides and cosmogenic radionuclides. They lead to external and internal radiation exposure. This study focused on levels of radioactivity due terrestrial radionuclides. The radionuclides of interest are Uranium-238 and Thorium-232 and their decay series nuclides as well as Potassium-40.

The major sources of external gamma radiation are due to ^238^U, ^232^Th and their decay products and ^40^K. ^238^U and its daughters rather than ^226^Ra and its daughter products are responsible for the major fraction of the internal dose received by humans from naturally occurring radionuclides (de Oliveira et al. 2001). Even though the concentrations of these radionuclides are widely distributed in nature, they have been found to depend on the local geological conditions and as a result vary from place to place (Xinwei et al. 2006). The specific levels are related to the type of rock from which the soil originates. Higher radioactivity levels are associated with igneous rocks such as granite and lower levels with sedimentary rocks. There are exceptions however, as some shales and phosphate rocks have relatively high content of radionuclides (Uosif 2007).

Among the natural radionuclides, alpha and beta emitters are considered the most important with respect to potential internal radiation exposure to humans particularly through ingestion of food and water (UNSCEAR 2000) as approximately 20 % of Ra isotopes and 10–15 % of the Pb isotopes which are decay products of ^238^U and ^232^Th considered for this work reaches the blood stream and are distributed to the whole body and follows the same metabolism as calcium (UNSCEAR 1988). The percentage distribution of annual intakes of Uranium and Thorium series radionuclides in diet ranges between 4 and about 96 % (UNSCEAR 2000), as a result accumulation of these radionuclides through the ingestion have significant health effects such as bone cancer, leukemia and increase in blood pressure (Tettey-Larbi et al. 2013).

Mining has been identified as one of the potential sources of exposure to naturally occurring radioactive materials (UNSCEAR 2000). As a result it is necessary for baseline studies to be carried out prior to the commencement of mining activity and subsequently similar studies be conducted to ascertain the levels of these radionuclides being turned out as a result of the mining activities in the operational phase of the mine.

Perseus Mining (Ghana) Ltd., is one of the several mining companies in Ghana located at Ayanfuri, of Ghana. Earlier study by Faanu et al. (2013, 2014), on part of the mine’s concession gave an average activity concentrations for ^238^U, ^232^Th and ^40^K as 64.3, 68.4 and 1243.9 Bqkg^−1^ respectively. This study became necessary as a result of operational expansion to another part of the concession, hence, the need to carried baseline radioactivity survey in order to establish the levels of radioactivity in the area before commencement. This will also help to determine whether or not the operational expansion of the mines to these new concession it operations will have added or raised the levels of NORMs in and round the study area in a future operation and post operation of the facility. The new concession is dotted along and within five main towns namely; Gyaman, Odumkrom, Wampem, Ayanfuri and Nkonya which could be affected as a result of the mine’s operations.

Therefore, the main objective of this study was to measure and assess the baseline radioactivity levels at the new concession of the mine as well as the immediate surroundings so that reference data could be established before the mine starts processing of the gold ore in the area. The study focused on determination of activity concentration and distribution of naturally occurring radionuclides of U/Th decay series and ^40^K in soil, rock, and ore samples by gamma spectrometry and gross alpha and gross beta activities in water samples. For the gross alpha and gross beta activities in the water samples, particular attention was focused on the major alpha and beta emitting radionuclides in the Uranium and Thorium Series which are of importance to internal irradiation. Lasheen et al. (2008), recognizes ^238^U, ^234^U, ^230^Th, ^226^Ra, ^210^Pb and ^210^Po for the Uranium series and ^232^Th, ^228^Ra and ^228^Th for the Thorium series as the major alpha and beta emitting radionuclides respectively.

## Description and local geology of study area

The study area is the eastern concession of Perseus Mining (Ghana) Limited and its immediate surroundings up to about 20 km from the plant site. The mine is bounded by two districts namely; the Upper Denkyira East and Amenfi West districts. The goldmine is located at latitude 5°57′21.45″N and longitude 1°54′35.35″W. Within this concession, six gold deposits open pits namely; Chirawewa, Bokiti, Small Fetish, Big Fetish, Esuajah North and Esuajah South will be mined. Surrounding these open pits are the following towns: Gyaman, Odumkrom, Wampem, Ayanfuri and Nkonya.

**Fig. 1:**
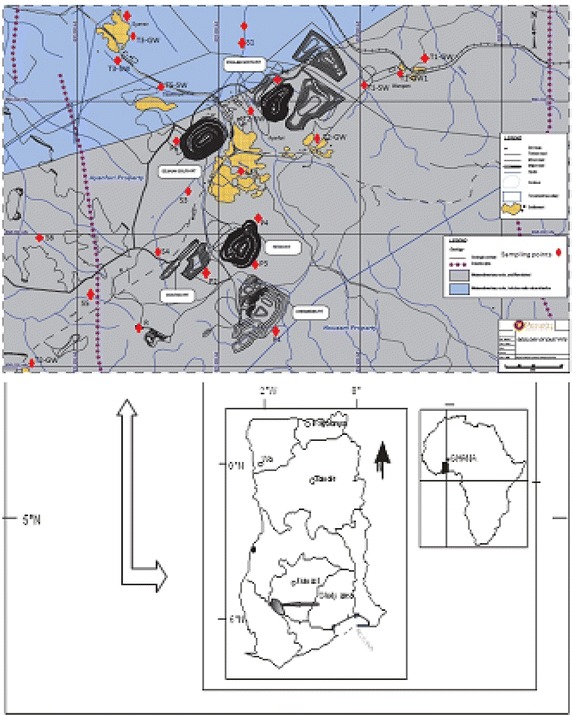
Geological map of the study area

The gold ore in the area occurs both in classic Ashanti-style sediment of shear zones and with granitic plugs and sills or dykes situated along two or three regional shear structures. In excess of 24 gold occurrences exist in the Ayanfuri property of which granitic intrusive host majority of these and more than 80 % of the known gold resource. While the later deposits formed predominantly ductile regime with generally discontinuous, pinch and swell higher grade gold shoots, the granite hosted occurrences developed in a brittle rock and found to be significantly broader with more evenly distributed, though lower grade gold tenor. Figure 1 is the geological map of the study area with sampling points in red dots.

## Methods

### Sampling and sample preparation for gamma spectrometry analysis

A total of 30 samples were randomly collected within selected areas of the new concession and the surrounding communities. They included 14 soil, rock and ore samples and 16 water samples.

In the laboratory, each of the soil, rock and ore samples were air dried on trays for 7 days and then oven dried at a temperature of 105 °C for between 3 and 4 h until all moisture was completely lost. The dried samples were then grinded into fine powder using a stainless steel ball mill and sieved through a 2 mm mesh size and poured into 1 l Marinelli beakers. The Marinelli beakers were hermetically sealed, weighed and stored for 4 weeks, to allow the short-lived daughters of ^238^U and ^232^Th decay series to attain equilibrium with their long-lived parent radionuclides (ASTM 1983, 1986). The soil samples were each counted using a sodium iodide detector for a period of 72,000 s (20 h).

For the water samples, 500 ml of each of the water samples was acidified with 1 ml of concentrated HNO_3_ and evaporated to near dryness on a hot plate in a fume hood. The residue in the beaker was rinsed with 1 M HNO_3_ and evaporated again to near dryness. The residue was dissolved in minimum amount of 1 M HNO_3_ and transferred into a weighed 25 mm stainless steel planchet. The planchet with its content was heated until all moisture had evaporated. It was then stored in a desiccator and allowed to cool and prevented from absorbing moisture. The water samples were each counted using the low background Gas-less Automatic Alpha/Beta counting system (Canberra iMatic™) for a period of 12,000 s (200 min or 3.33 h).

### Instrumentation and calibration

The soil, rock and ore samples measurement were made by direct non-destructive instrumental analysis with a computerized gamma spectrometry system made up of NaI(TI) detector and measuring assembly. The specifications of the detector system used for this study are as follows: cylindrical scintillation detector Model 3M3/3-X, Serial number ETI 9305 having a 1.2″ × 1.2″ end window, manufactured by Saint-Gobain Crystals, USA. The detector system consists of a vertically sealed assembly which includes the NaI(TI) crystal and is coupled to ORTEC Multichannel Buffers (MCBs) for data acquisition and processing using a MAESTRO^®^-32 software program. A high voltage supply provides the appropriate bias to the detector system. The conversion gain of the detector is up to 1024 channels. In order to reduce background gamma radiation from the room in which the detector is installed in, a locally fabricated cylindrical lead shield (20 mm thickness) with a fixed bottom and a movable lid to shield the detector. Within the lead shield are also copper, cadmium and plexiglass (3 mm thickness each) to absorb X-rays and other photons that might be produced in the lead. The ambient temperature around the detector varied between 20 and 27 °C during the period of measurement. The identification of individual radionuclides was performed using their gamma ray energies and the quantitative analysis of radionuclides was performed using gamma ray spectrum analysis software, ORTEC MAESTRO-32.

Before the analysis of soil/rock-ore samples, energy and efficiency calibration were carried out using liquid mixed standard radionuclide solution supplied by the IAEA with volume and density of 1000 ml and 1.0 g^−3^ respectively. The energy and efficiency calibrations were carried out by counting standard radionuclides of known activities with well defined energies in the energy range of 60 to ~2000 keV. The background spectrum was also used to determine the minimum detectable activities of ^238^U (0.34 Bq), ^232^Th (0.33 Bq), and ^40^K (1.62 Bq) at the 95 % confidence level.

The low background gas-less automatic alpha/beta counting system (Canberra iMatic™) calibrated with alpha (^241^Am) and beta (^90^Sr) standards was used to count the prepared water samples. The system uses a solid state passivated implanted planar silicon (PIPS) detector for alpha and beta detection. The alpha and beta efficiencies were determined to be 36.39 ± 2.1 and 36.61 ± 2.2 % respectively. The background readings of the detector for alpha and beta activity concentrations were respectively 0.04 ± 0.01 and 0.22 ± 0.03 cpm.

### Calculation of activity concentration and estimation of doses

#### Soil, rock and ore samples

The activity concentration of ^238^U was calculated from the average peak energies of 351.92 keV of ^214^Pb, and 609.31 keV of ^214^Bi. Similarly, the activity concentration of ^232^Th was determined from the average energies of 238.63 keV of ^212^Pb and 911.21 keV of ^228^Ac. The activity concentration of ^40^K was determined from the energy of 1460.83 keV. The analytical expression used in the calculation of the activity concentrations in Bqkg^−1^ is as shown in Eq. (1) (Ebaid 2010)

1 $$A_{sp} = \frac{{N_{D} }}{{p \cdot T_{c} \cdot \eta (E) \cdot m}}$$

where N_D_ is the net counts of the radionuclide in the samples, *p* is the gamma ray emission probability (gamma ray yield), η(E) is the absolute counting efficiency of the detector system, T_c_ is the sample counting time, m is the mass of the sample (kg).

The external gamma dose rate (D_γ_) at 1.0 m above ground for the soil/rock-ore pad-ore pad samples was calculated from the activity concentrations using Eq. (2) (Uosif 2007).

2 $$D_{\gamma } \,({\text{nGyh}}^{ - 1} ) = DCF_{K} \times A_{K} + DCF_{U} \times A_{U} + DCF_{Th} \times A_{Th}$$

where DCF_K_, DCF_U_, DCF_Th_ are the absorbed dose rate conversion factors for ^40^K, ^238^U and ^232^Th in nGy h^−1^ Bqkg^−1^ and A_K_, A_u_ and A_Th_ are the activity concentrations for ^40^K, ^238^U and ^232^Th respectively

$$\begin{aligned} & {\text{DCF}}_{\text{K}} = 0.0417\,{\text{nGy}}/{\text{h}}/{\text{Bqkg}}^{ - 1} ;\quad {\text{DCF}}_{\text{U}} = 0.462\,{\text{nGy}}/{\text{h}}/{\text{Bqkg}}^{ - 1} ; \\ & {\text{DCF}}_{\text{Th}} = 0.604\,{\text{nGy}}/{\text{h}}/{\text{Bqkg}}^{ - 1} \\ \end{aligned}$$

The average annual effective dose was calculated from the absorbed dose rate by applying the dose conversion factor of 0.7 SvGy^−1^ and an outdoor occupancy factor of 0.2 (UNSCEAR 2000) represented by Eq. (3)

3 $$E_{\gamma } = D_{r} \times 0.2 \times 8760 \times 0.7$$

where E_γ_ is the average annual effective dose and D_γ_ is the absorbed dose rate in air.

For comparative analysis, similar average outdoor external gamma dose rate were determined by taking an average of five measurements of the ambient gamma dose rates at 1 m above the ground of sampling in μGyh^−1^ with a radiation survey meter. The annual effective dose (E_γ,ext_) was then estimated from the measured average outdoor external gamma dose rate from the Eq. (4)

4 $$E_{\gamma ,ext} = D_{\gamma ,ext} T_{\exp } DCF_{ext}$$

where D_γ,ext_ is the average outdoor external gamma dose rate μGyh^−1^, T_exp_ is the exposure duration per year, 8760 h and applying an outdoor occupancy factor of 0.2, DCF_ext_ is the effective dose to absorbed dose conversion factor of 0.7 SvGy^−1^ for environmental exposure to gamma rays (UNSCEAR 2000).

The radiological risk of NORM in soils in the study area which may be used as building materials was assessed by calculating the radium equivalent activity (Ra_eq_) and the external hazard and internal hazard indices. The Ra_eq_ is a widely used hazard index and it was determined using Eq. (5) (Xinwei et al. 2006):

5 $$Ra_{eq} = C_{Ra} + 1.43C_{Th} + 0.077C_{K}$$

where C_Ra_, C_Th_ and C_K_ are the activity concentrations of ^226^Ra, ^232^Th and ^40^K respectively. In its application, the definition of Ra_eq_, it is assumed that 370 Bqkg^−1^ of ^226^Ra, 259 Bqkg^−1^ of ^232^Th and 4810 Bqkg^−1^ of ^40^K produce the same gamma ray dose rate. The above criterion only considers the external hazard due to gamma rays in building materials.

Another criterion used to estimate the level of gamma ray radiation associated with natural radionuclides in specific construction materials is defined by the terms External hazard index (H_ex_) and Internal hazard index (H_in_) as shown in Eqs. (6) and (7) (OECD/NEA 1979; Beretka and Mathew 1985; Alam et al. 1999; Higgy et al. 2000)

6 $$H_{ex} = \frac{{C_{Ra} }}{370} + \frac{{C_{Th} }}{259} + \frac{{C_{K} }}{4810}$$

where C_Ra_, C_Th_ and C_K_ are the activity concentrations of ^226^Ra, ^232^Th and ^40^K respectively. The value of the external hazard index must be less than unity for the external gamma radiation hazard to be considered negligible.

The internal hazard index (H_in_) due to Radon and its daughters were calculated from Eq. (7). This is based on the fact that, Radon and its short-lived products are also hazardous to the respiratory organs

7 $$H_{in} = \frac{{C_{Ra} }}{185} + \frac{{C_{Th} }}{259} + \frac{{C_{K} }}{4810}$$

where C_Ra_, C_Th_ and C_K_ are the activity concentrations of ^226^Ra, ^232^Th and ^40^K respectively. For construction materials to be considered safe for construction of dwellings, the internal hazard index should be less than unity.

#### Water samples

The activity concentration of both gross alpha and gross beta was determined using the expression in Eq. (8) (Tettey-Larbi et al. 2013):

8 $$A_{\alpha /\beta } = \frac{Activity}{{W_{Vol} }}$$

where *A*_*α*/*β*_ is the activity concentration of gross alpha or gross beta in Bql^−1^ and *W*_*Vol*_ is the volume of the water sample in litres. The activity of alpha or beta in Bq was obtained by subtracting the background activity of both gross alpha and gross beta from the total activity of the sample. The average annual alpha or beta committed effective dose for a particular water sample was determined by averaging the individual annual committed effective doses contributed by the major alpha or beta emitters in the ^238^U and ^232^Th series of the naturally occurring radionuclides as shown in Eq. (9) (Tettey-Larbi et al. 2013):

9 $$E_{ing, w} (\alpha /\beta ) = \frac{{I_{w} }}{{W_{Vol} \cdot N_{R} (\alpha /\beta )}}\mathop \sum \limits_{i}^{R(\alpha /\beta )} A_{\alpha /\beta } \times DCF_{ing} (\alpha /\beta )$$

where *E*_*ing*,*w*_(*α*/*β*) is the average gross annual alpha or beta committed effective dose in the water sample, *A*_*α*/*β*_ is the gross alpha or beta activity concentration in the water sample in Bql^−1^, *I*_*w*_ is the consumption rate for the intake of the water of 730 l year^−1^ (WHO 2004), *W*_*Vol*_ is the volume water used for the analysis, *N*_*R*_(*α*/*β*) is the number of radionuclides considered as major alpha or major beta emitters in the ^238^U and ^232^Th series of the naturally occurring radionuclides and *DCF*_*ing*_(*α*/*β*) is the ingestion dose coefficient in Sv/Bq of the natural radionuclides from UNSCEAR report (UNSCEAR 2000).

### Total annual effective dose

The total annual effective dose (E_T_) to members of the public was calculated using ICRP dose calculation method (ICRP 1991, 2007). The analytical expression for the total effective dose is provided in Eq. (10)

10 $$E_{T} = E_{\gamma } (U,Th,K) + E_{ing,w} (\alpha /\beta )$$

where E_T_ is the total effective dose in Sievert (Sv), E_γ_ (U, Th, K) is the external gamma effective dose from the soil/rock-ore pad samples, E_ing,w_ (α/β) is the effective dose from the consumption of water due to gross alpha and gross beta activity concentrations.

## Results and discussion

The list of sample location index with coordinates is given in Table 1 along with remarks for soil, water and ore, respectively.

**Table 1:** Sampling location index and co-ordinates

Location index and descriptions		Co-ordinates	
Location index	Description	Latitude	Longitude
P_1_	Open pit named Chirawewa	5°56′25.17″N	1°53′29.58″W
P_2_	Open pit named Bokiti	5°56′53.43″N	1°54′08.68″W
P_3_	Open pit named Small Fetish	5°57′05.83″N	1°53′39.36″W
P_4_	Open pit named Big Fetish	5°56′53.43″N	1°54′09.68″W
P_5_	Open pit named Esuajah North	5°58′07.76″N	1°53′31.31″W
P_6_	Open pit named Esuajah South	5°57′50.95″N	1°54′01.20″W
T_1_-SW	Wampem community surface water	5°58′20.39″N	1°52′46.33″W
T_1_-GW	Wampem community ground water	5°58′27.89″N	1°52′30.58″W
T_1_-GW_2_	Wampem community ground water	5°58′25.68″N	1°52′21.02″W
T_2_-SW	Ayanfuri community surface water	5°57′57.79″N	1°53′41.25″W
T_2_-GW	Ayanfuri community ground water	5°58′03.74″N	1°53′47.56″W
T_3_-SW	Gyaman community surface water	5°58′30.08″N	1°54′36.22″W
T_3_-GW	Gyaman community surface water	5°58′49.17″N	1°54′46.09″W
T_4_-GW	Nkonya community ground water	5°55′51.24″	1°55′26.92″
T_5_-SW	Odumkrom community surface water	5°59′10.91″N	1°53′31.70″W
S_1_	Stream within the mine concession	5°56′51.17″N	1°54′19.13″W
S_2_	Stream within the mine concession		
S_3_	Stream within the mine concession	5°56′09.51″N	1°55′18.59″W
S_4_	Stream within the mine concession		
S_5_	Stream within the mine concession	5°59′08.46″N	1°53′20.53″W
S_6_	Stream within the mine concession	5°56′34.09″N	1°54′15.72″W
R	Residence of mine workers	5°56′16.69″N	1°54′18.90″W
R-GW_1_	Borehole at the residence		
R-GW_2_	Borehole at the residence		
O_7_	An ore from Bokiti pit		
O_8_	An ore from Fetish pit		

**Fig. 2:**
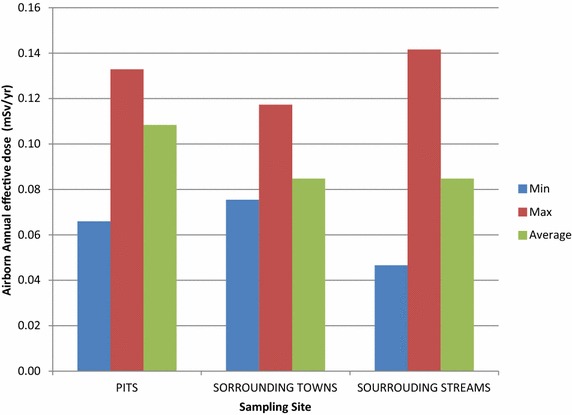
Comparison of annual effective doses from different sampling site due to airborne gamma exposure pathways of radiation

Table 2 shows the absorbed dose rate measured in air at 1 m above the ground at the soil/rock-ore pad and water sampling points in the study area and its surrounding communities. The measured average absorbed dose rate was determined to be 0.08 ± 0.02 μGyh^−1^ (80 ± 20 nGyh^−1^). The corresponding average annual effective dose was calculated to be 0.093 ± 0.028 mSv (93 ± 28 μSv) in a range of 0.047–0.142 mSv (49–142 µSv). According to UNSCEAR (2000) report, the worldwide average absorbed dose rate in air measured from terrestrial gamma radiation is 60 nGyh^−1^ (0.059 μGyh^−1^). By comparison, the results of the absorbed dose rates in this study compare well with the range of dose rates values reported for other countries (UNSCEAR 2000) as well as results from similar studies carried out in other mines in Ghana (Darko et al. 2010; Faanu et al. 2010, 2013, 2014) although the average absorbed dose rate of 80 ± 20 nGyh^−1^ measured in air from the study area is above the worldwide average. The reasons for the higher values of the doses for external gamma could be due to difference in geological formations as well as contribution from cosmogenic radionuclides in addition to terrestrial radionuclides as observably in Fig. 2 the pits give the highest average effective dose although the maximum value was recorded around the streams.

**Table 2:** Average absorbed dose rate in air at 1 m above sampling points in the study areas and calculated annual effective dose

Sampling location	Absorbed dose rate (μGyh^−1^)		Annual effective dose (mSv)
	Range	Average ± σ	
P_1_	0.04–0.14	0.08 ± 0.03	0.093
P_2_	0.04–0.17	0.10 ± 0.05	0.124
P_3_	0.04–0.13	0.09 ± 0.03	0.109
P_4_	0.06–0.14	0.10 ± 0.03	0.126
P_5_	0.09–0.12	0.11 ± 0.01	0.133
P_6_	0.02–0.10	0.05 ± 0.03	0.066
T_1_	0.06–0.14	0.10 ± 0.03	0.117
T_2_	0.04–0.09	0.06 ± 0.02	0.076
T_3_	0.04–0.09	0.06 ± 0.02	0.078
T_4_	0.04–0.08	0.06 ± 0.02	0.075
T_5_	0.03–0.09	0.06 ± 0.02	0.078
S_1_	0.04–0.13	0.09 ± 0.03	0.112
S_2_	0.08–0.16	0.12 ± 0.03	0.142
S_3_	0.03–0.08	0.06 ± 0.02	0.067
S_4_	0.02–0.06	0.04 ± 0.02	0.047
S_5_	0.03–0.10	0.06 ± 0.02	0.075
S_6_	0.04–0.08	0.05 ± 0.02	0.066
Average ± σ	0.02–0.17	0.08 ± 0.02	0.093 ± 0.028

σ—Standard deviation

Table 3 shows the activity concentrations of ^238^U, ^232^Th and ^40^K in the soil/rock-ore pad samples. The average value of the activity concentrations of ^238^U is 65.1 ± 2.2 Bqkg^−1^ in a range of 29.0–97.0 Bqkg^−1^. For ^232^Th, the average activity concentration is 71.8 ± 2.2 Bqkg^−1^ in range of 35.0–116.7 Bqkg^−1^ and that of ^40^K is 1168.3 ± 15.8 Bqkg^−1^ in a range of 500.0–1795.9 Bqkg^−1^. The results of this study also compare with the previous study that was carried in Perseus Mining (Ghana) Ltd., (Fannu et al. 2013, 2014). As shown in Table 3, the average values of the activity concentrations of ^238^U and ^232^Th in this study are about two times higher than the world average whilst that of ^40^K is about three times higher than values in normal continental soils (UNSCEAR 2000). The high activity concentration of ^40^K is because the rock ore of the mine is associated feldspar which belongs to a group of hard crystalline minerals that consist of aluminium silicates of potassium, sodium, calcium or barium. Even though the average values in this study are higher than the worldwide average values, activity concentrations are still far below the exemption values of 1000 Bqkg^−1^ for ^238^U and ^232^Th and 100,000 Bqkg^−1^ for ^40^K in materials that will warrant regulatory control (IAEA 1996).

**Table 3:** Average activity concentrations of ^238^U, ^232^Th and ^40^K in soil, rock and ore pad samples in the study area

Sample location	Activity concentration (Bqkg^−1)^		
	^238^U	^232^Th	^40^K
P_1_	97.0 ± 2.4	70.8 ± 2.4	1795.9 ± 17.8
P_2_	72.0 ± 2.3	84.2 ± 2.2	1445.4 ± 16.3
P_3_	86.8 ± 2.1	64.4 ± 2.1	1317.2 ± 17.5
P_4_	56.3 ± 2.0	61.8 ± 2.2	1722.4 ± 14.6
P_5_	62.2 ± 3.8	116.7 ± 3.0	1445.0 ± 20.4
P_6_	74.3 ± 2.3	89.6 ± 2.4	1544.1 ± 16.8
O_7_	62.1 ± 2.1	61.6 ± 2.2	1470.6 ± 17.2
O_8_	29.0 ± 1.6	35.0 ± 1.6	748.3 ± 12.0
S_1_	66.4 ± 1.9	81.6 ± 2.1	1241.2 ± 15.0
T_1_	69.2 ± 2.6	76.1 ± 2.1	844.8 ± 14.9
T_2_	70.0 ± 1.9	83.7 ± 2.0	720.8 ± 14.6
T_3_	57.0 ± 2.1	63.9 ± 1.9	500.0 ± 13.9
T_4_	61.3 ± 2.1	68.7 ± 2.0	749.5 ± 14.6
T_5_	48.4 ± 1.9	47.1 ± 2.1	811.3 ± 15.1
Range	29.0–97.0	35.0–116.7	500.0–1795.9
Average ± σ	65.1 ± 2.2	71.8 ± 2.2	1168.3 ± 15.8
World average	35	30	400

σ—Standard deviation

**Fig. 3:**
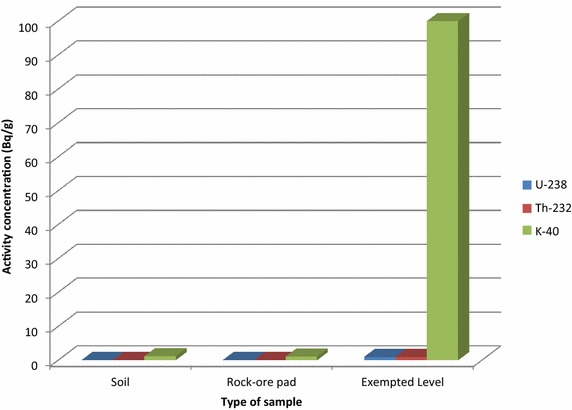
Comparison of the activity concentrations of ^238^U, ^232^Th and ^40^K in different samples with exempt levels recommended in the Basic Safety Standard (IAEA 1996)

A comparison of the activity concentrations of the radionuclides in soil and rock-ore pad with exemption levels recommended in the Basic Safety Standards (IAEA 1996) are shown in Fig. 3. The activity concentrations of the radionuclide in the different types of samples are quite uniform and do not show any significant variation.

The average gamma dose rate and annual effective dose from terrestrial gamma rays calculated from soil/rock-ore pad activity concentrations are shown in Table 4. The average absorbed dose rate was calculated to be 741.6 ± 260.1 nGyh^−1^ with ranges which are by factors of 6–16 higher than the dose rate measured in air at 1 m above the ground. The average absorbed dose rate due to the soil concentrations is also about 12 times higher than the worldwide average value of 60 nGyh^−1^ (UNSCEAR 1993, 2000). This difference could be attributed to vast differences in geology and geochemical state of the sampling sites. The corresponding average annual effective dose estimated from the soil concentrations is 0.91 ± 0.32 mSv.

**Table 4:** Absorbed dose rates, radium equivalent activity, external and internal hazard and annual effective doses due to ^238^U, ^232^Th and ^40^K in soil and ore pad samples in the study area

Sample location	Absorbed dose rate (nGyh^−1^)	Radium equivalent activity (Bqkg^−1^)	External hazard index	Internal hazard index	Annual effective dose (mSv)
P_1_	1121.5	336.5	0.9	1.2	1.38
P_2_	914.9	303.7	0.8	1.0	1.12
P_3_	829.0	280.2	0.8	1.0	1.02
P_4_	1071.2	277.4	0.7	0.9	1.31
P_5_	929.3	340.3	0.9	1.1	1.14
P_6_	977.1	321.3	0.9	1.1	1.20
O_7_	919.3	263.3	0.7	0.9	1.13
O_8_	469.3	136.6	0.4	0.4	0.58
S_1_	790.2	278.6	0.8	0.9	0.97
T_1_	548.3	243.1	0.7	0.8	0.67
T_2_	477.0	245.2	0.7	0.9	0.58
T_3_	333.9	187.0	0.5	0.7	0.41
T_4_	487.0	217.3	0.6	0.8	0.06
T_5_	513.8	178.2	0.5	0.6	0.63
Range	333.9–1121.5	136.6–340.3	0.4–0.9	0.4–1.2	0.41–1.38
Average ± σ	741.6 ± 260.1	257.8 ± 61.1	0.7 ± 0.2	0.9 ± 0.2	0.91 ± 0.32

σ—Standard deviation

Table 4 also shows the results of the hazard assessment of soil/rock-ore pad with respect to radium equivalent (Ra_eq_) activity in Bqkg^−1^, external hazard (H_ex_) and the internal hazard (H_in_) indices. Radiological hazard assessment of natural radioactivity in building materials is usually determined from the activity concentrations of ^226^Ra, ^232^Th and ^40^K. ^238^U is replaced by ^226^Ra because 98.5 % of the radiological hazard of uranium-series is due to radium and its decay products. The maximum value of Ra_eq_, in building materials must be <370 Bqkg^−1^ for the material to be considered safe for use. The external and internal hazard indices must also be less than unity in order to keep the radiation hazard insignificant. This implies that, the average external radiation exposure due to the radioactivity from these radionuclides in materials to be used for constructions must be limited to 1.5 mSv year^−1^ (OECD/NEA 1979; Beretka and Mathew 1985). The average value of the radium equivalent activity in this study is 257.8 ± 61.1 Bqkg^−1^ in a range of 136.6–340.3 which is below the recommended limit of 370 Bqkg^−1^. The calculated H_ex_ in the soil/rock-ore pad samples ranged from 0.4 (Big Fetish Pit Ore pad) to 0.9 (soil sample from Chirawewa Pit and Esuajah North and South Pit) and an average value of 0.7 ± 0.2 for the study area. Similarly for the H_in_, the values ranged from 0.4 (Big Fetish Pit Ore pad) to 1.2 (Chirawewa Pit soil) with an average of 0.9 ± 0.2. For the internal hazard 21.4 % of the samples had values exceeding the recommended limit of 1.0 which implies that these materials if used for building purposes could be a source of internal hazard due to Radon and its progeny. While 78.6 % of the samples have values either exactly at the recommended limit or below the recommended limit. In general however, the annual effective doses calculated from the various samples are considered insignificant in respect to the annual construction limit of 1.5 mSv year^−1^.

The activity concentrations of gross-α and gross-β in water samples from the pits, surface water and underground water (bore holes) used in the surrounding communities of the study area and their corresponding committed effective dose are shown in Table 5. Radionuclide concentrations in groundwater depend on the dissolution of minerals from rock aquifers. The activity concentrations of gross-α in the water samples varied in a range of 0.0004 Bql^−1^ in surface water at Wampem to 0.0075 Bql^−1^ in surface water in Nkonya borehole with a corresponding average annual committed effective dose of 0.0007 ± 0.0005 mSv. For the gross-β, the activity concentrations varied in a range of 0.0104 Bql^−1^ for water taken from a stream in Odumkrom to 0.0452 Bql^−1^ for water from the raw underground water at the camp site with a correspond average annual committed effective dose of 0.0170 ± 0.0042 mSv. The average committed annual effective dose due to both gross alpha and beta was estimated to be 0.0089 ± 0.0023 mSv. All the water sources had gross-α and gross-β values below the WHO screening levels for drinking water at no further action is required. This indicates that all the water sources in the study area which are designated for drinking and domestic purposes do not have significant natural radioactivity.

**Table 5:** Gross-α and gross-β activity concentrations (Bql^−1^) and their corresponding committed annual effective dose in water samples from the Pits and surrounding communities

Sample location	Type of water	Activity concentration (Bql^−1^)		Committed annual effective dose for (mSv year^−1^)	
		Gross alpha	Gross beta	Gross alpha	Gross beta
P_1_	Surface water	0.0013	0.0367	0.0003	0.0185
P_2_	Surface water	0.0042	0.0430	0.0009	0.0217
P_3_	Surface water	0.0011	0.0389	0.0002	0.0196
P_4_	Surface water	0.0019	0.0357	0.0004	0.0180
P_5_	Surface water	0.0011	0.0391	0.0002	0.0197
T_1_-SW	Surface water	0.0004	0.0319	0.0001	0.0161
T_2_-SW	Treated water	0.0067	0.0311	0.0015	0.0157
T_3_-SW	Surface water	0.0010	0.0390	0.0002	0.0196
T_5_-SW	Surface water	0.0020	0.0104	0.0004	0.0052
S_1_	Surface water	0.0034	0.0310	0.0007	0.0156
T_1_-GW	Ground water	0.0013	0.0314	0.0003	0.0158
T_2_-GW	Ground water	0.0058	0.0392	0.0013	0.0197
T_3_-GW	Ground water	0.0015	0.0243	0.0003	0.0122
T_4_-GW	Ground water	0.0075	0.0354	0.0016	0.0178
R-GW_1_	Ground water	0.0065	0.0452	0.0014	0.0228
R-GW_2_	Ground water	0.0050	0.0289	0.0011	0.0146
Range		0.0004–0.0075	0.0104–0.0452	0.0001–0.0016	0.0052–0.0228
Average		0.0032 ± 0.0024	0.0338 ± 0.0083	0.0007 ± 0.0005	0.0170 ± 0.0042
Total average				0.0089 ± 0.0023	
GSB recommended limit		0.1000	1.0000		
WHO recommended limit		0.5000	1.0000		

σ—Standard deviation, GSB—Ghana Standards Board, WHO—World Health Organisation

**Fig. 4:**
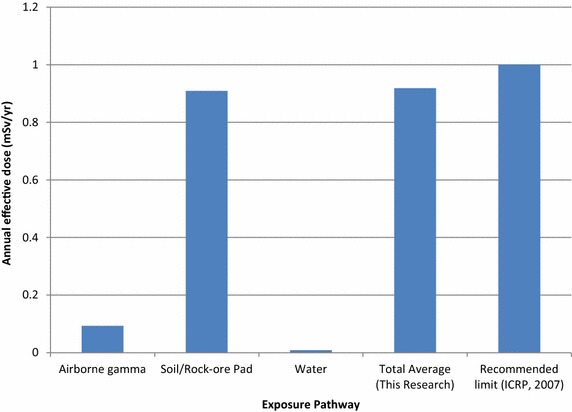
Comparison of annual effective doses from different exposure pathways of radiation with their corresponding total effective dose compared with the recommended limit by ICRP

Figure 4 shows the comparison of the results of the annual effective doses due to ^238^U, ^232^Th and ^40^K calculated from the airborne gamma radiation and soil/rock-ore pad samples. Figure 4 shows that the annual effective doses due to to ^238^U, ^232^Th and ^40^K in the soil/rock-ore pads were higher than that due to the external gamma dose rates. The reasons for the higher values of the doses for soil/rock-ore pads could be due to contribution from the high concentration of terrestrial radionuclides especially ^40^K since is a purely gamma emitter. It also compares the total annual effective dose to the recommended limit of 1 mSv for the public.

## Conclusions

The study considered public exposure in the mining environment due to two exposure pathways; namely direct external gamma ray exposure from natural radioactivity concentrations in soil/rock-ore pads radioactivity due to ^238^U, ^232^Th and ^40^K and internal exposure due to natural radioactivity by accessing the activity concentrations of gross-α and gross-β in water samples from the pits, surface water and underground water (bore holes) used in the surrounding communities of the study area.

The average activity concentrations of ^238^U, ^232^Th and ^40^K in the soil and rock-ore pad samples were calculated to be 65.1 ± 2.2, 71.8 ± 2.2 and 1168.3 ± 15.8 Bqkg^−1^ respectively. The results in this study compared well with studies carried out in Ghana and other countries and with the worldwide average activity concentrations (UNSCEAR 2000; Darko et al. 2010; Faanu et al. 2010, 2013, 2014; Huang et al. 2014). The average annual effective doses estimated from direct external gamma-ray exposure from natural radioactivity at 1 m above sampling area and that due to activity concentrations of ^238^U, ^232^Th and ^40^K in the soil and rock-ore pad samples were 0.093 ± 0.028 and 0.91 ± 0.32 mSv respectively. In order to assess whether the soil/rock-ore pad in the study area could be a source of public radiation exposure if used for building purposes, the following hazard indices were determined; radium equivalent (Ra_eq_) activity in Bq/kg, external (H_ex_) and the internal hazard (H_in_). The average value of the radium equivalent activity in this study is 257.8 ± 61.1 Bqkg^−1^ which is below the recommended limit of 370 Bqkg^−1^. The mean external and internal indices were 0.7 ± 0.2 and 0.9 ± 0.2 respectively where also below the recommended limit of 1. Therefore the annual effective doses calculated from the various samples are considered insignificant in respect to the annual construction limit of 1.5 mSv year^−1^.

In general the results in this study are comparable to similar studies carried out previously at the mines and in other mines in Ghana (Darko et al. 2010; Faanu et al. 2010, 2013, 2014) as well as in other countries (UNSCEAR 2000; Huang et al. 2014). It also indicates insignificant levels of the natural radionuclides in the study area and therefore radiation exposure to workers as well as members of the public is not of any significant hazard and the level risk is considered generally insignificant. Finally it also implies that previous mining activities had not imparted negatively in terms of radiological hazard to the communities in this area.

## References

[CR1] Alam MN, Miah MMH, Chowdhury MI, Kamal M, Ghose S, Islam MN, Mustafa MN, Miah MSR (1999). Radiation dose estimation from radioactivity analysis of lime and cement used in Bangladesh. J Environ Radioact.

[CR2] ASTM (1983) Standard Method for sampling surface soils for radionuclides. American Society for Testing Materials, report no. C. ASTM, Philadelphia, pp 983–998

[CR3] ASTM (1986) Recommended practice for investigation and sampling soil and rock for engineering purposes. In: Annal book of ASTM standards (04/08). American Society for Testing Materials, Report No. D, 420. ASTM, Philadelphia, pp 109–113

[CR4] Beretka J, Mathew PJ (1985). Natural radioactivity of Australian building materials, industrial wastes and by-products. Health Phys.

[CR5] Darko EO, Faanu A, Razak A, Emi-Reynolds G, Yeboah J, Oppon OC, Akaho EHK (2010). Public exposure hazards associated with natural radioactivity in open-pit mining in Ghana. Radiat Prot Dosim.

[CR6] De Oliveira J, Paci Mazzilli B, da Costa P, Akiko Tanigava P (2001). Natural radioactivity in Brazilian bottled waters and consequent doses. J Radioanal Nucl Chem.

[CR7] Ebaid YY (2010). Use of gamma-ray spectrometry for uranium isotopic analysis of environmental samples. Rom J Phys.

[CR8] Faanu A, Ephraim JH, Darko EO (2010). Assessment of public exposure to naturally occurring radioactive materials from mining and mineral processing activities of Tarkwa Goldmine in Ghana. Environ Monit Assess.

[CR9] Faanu A, Kpeglo DO, Sackey M, Darko EO, Emi-Reynolds G, Lawluvi H, Awudu R, Adukpo OK, Kansaana C, Ali ID, Agyeman B, Agyeman L, Kpodzro R (2013). Natural and artificial radioactivity distribution in soil, rock and water of the Central Ashanti Gold Mine.

[CR10] Faanu A, Lawluvi H, Kpeglo DO, Darko EO, Emi-Reynolds G, Awudu AR, Adukpo OK, Kansaana C, Ali ID, Agyeman B, Agyeman L, Kpodzro R (2014). Assessment of natural and anthropogenic radioactivity levels in soils, rocks and water, in the vicinityof chirano gold mine in Ghana. Radiat Prot Dosim.

[CR11] Higgy RH, El-Tahawy MS, Abdel-Fattah AT, Al-Akahawy UA (2000). Radionuclide content of building materials and associated gamma dose rates in Egyptian dwellings. J Environ Radioact.

[CR12] Huang YJ, Chen CF, Huang YC, Yue QJ, Zhong CM, Tan CJ (2014). Natural radioactivity and radiological hazards assessment of bone-coal from a vanadium mine in central China. Radiat Phys Chem.

[CR13] IAEA (1996) International basic safety standards for protection against ionising radiation and for the safety of radiation sources, safety series no. 115. IAEA, Vienna

[CR14] IAEA (2005) Naturally occurring radioactive materials (IV). In: Proceedings of the international conference held in Szczyrk, IAEA-TECDOC-1472, Poland

[CR15] ICRP (1991) 1990 recommendations of the International Commission on Radiological Protection. ICRP publication no. 60, Pergamon Press, Oxford 2053748

[CR16] ICRP (2007) 2006 recommendations of the International Commission on Radiological Protection. ICRP publication no. 103, Pergamon Press, Oxford 10.1016/j.icrp.2007.10.003 18082557

[CR17] Lasheen YF, Awwad NS, El-Khalafawy A, Abdel-Rassoul AA (2008). Annual effective dose and concentration levels of heavy metals in different types of tea in Egypt. Int J Phys Sci.

[CR18] OECD/NEA (1979). Exposure to radiation from natural radioactivity in building materials, report by NEA Group of Experts, Nuclear Energy Agency.

[CR19] Tettey-Larbi L, Darko EO, Schandorf C, Appiah AA, Sam F, Faanu A, Okoh DK, Lawluvi H, Agyeman BK, Kansaana C, Amoah PA, Osei RK, Agalga R, Osei S (2013). Gross alpha and beta activity and annual committed effective doses due to natural radionuclides in some medicinal plants commonly used in Ghana. Int J Sci Technol.

[CR20] UNSCEAR (1988) Sources, effects and risks of ionizing radiation. United Nations Scientific Committee on the effects of Atomic Radiation, 1988 report to the General Assembly, with annexes

[CR21] UNSCEAR (1993) Sources, effects and risks of ionizing radiation. United Nations Scientific Committee on the effects of Atomic Radiation. Exposures from natural sources of radiation, 1993 report to General Assembly, Annex A, New York

[CR22] UNSCEAR (2000) Sources, effects and risks of ionizing radiation. United Nations Scientific Committee on the effects of Atomic Radiation. Exposures from natural sources, 2000 report to General Assembly, Annex B, New York

[CR23] Uosif MAM (2007). Gamma-ray spectroscopic analysis of selected samples from the Nile river sediments in Upper Egypt. Radiat Prot Dosim.

[CR24] WHO (2004). Guidelines drinking-water quality.

[CR25] Xinwei L, Lingquig W, Xiaodan J, Leipeng Y, Gelian D (2006). Specific activity and hazards of Archeozoic–Cambrian rock samples collected from the Weibei area of Xhaanxi, China. Radiat Prot Dosim.

